# Clinical study on combining femtosecond thin- flap and LASIK with the Triple-A profile for high myopia correction

**DOI:** 10.1186/s12886-019-1115-0

**Published:** 2019-05-10

**Authors:** Kai Li, Chuan-Wei Zhang, De-Jian Hong, Jing Wu, Yi-Shuo Yao

**Affiliations:** Department of Ophthalmology, Affiliated Hospital of Nanjing University of Chinese Medicine,Nanjing, 155 Hanzhong Road, Nanjing, 210029 Jiangsu China

**Keywords:** High myopia, Laser refractive surgery, Visual acuity, Femtosecond LASIK, Triple-A procedure

## Abstract

**Background:**

Femtosecond laser–assisted LASIK (FS-LASIK) can make ultra-thin corneal flap accurately. MEL 90 excimer laser provides Triple-A ablation mode, which significantly reduces the amount of corneal tissue cutting. This study aimed to investigate the visual and refractive outcomes in patients with high myopia after thin-flap FS-LASIK using the 500 Hz pulse rate of the Triple-A profile.

**Methods:**

This prospective study included 90 eyes from 90 patients received thin-flap FS-LASIK using the 500 Hz pulse rate of the Triple-A profile. According to the pre-operative spherical equivalence (SE), the treated eyes were divided into two groups: the first group (ranged from − 9.0D to − 6.0D) and the second group (ranged from − 11.15D to − 9.0 D). The parameters evaluated pre-operatively and 6 month post-operatively included uncorrected distance visual acuity (UDVA), corrected distance visual acuity (CDVA), SE, efficacy and safety index, posterior central elevation, and corneal higher-order aberrations (HOAs).

**Results:**

The efficacy indexes were 1.149 ± 0.150 for the first group and 1.173 ± 0.136 for the second group (*P* > 0.05), whereas safety indexes were 1.135 ± 0.154 and 1.158 ± 0.137 (*P* > 0.05) respectively. Moreover, 93.8 and 90.6% of patients had an UDVA of 20/20, 51.2 and 49.8% had a UDVA of 20/16 for the first and second groups, respectively; yet, there were no significant differences between both groups at the 20/20 and the 20/16 levels (*P* > 0.05). 84 and 100% of the firse group patients had a SE within ±0.5 D and ± 1.0 D, and 82 and 100% of the second group patients. There was no significant myopia regression in both groups after 6 months follow-up. At 1, 3 and 6-month after surgery, there were no significant differences in the posterior central elevation between the two groups (*P* > 0.05). The induction of total HOAs, spherical aberration, and horizontal coma in the first group were significantly less than that in the second group at the 6- month follow-up (*P* < 0.05), while the differences of the RMS value of vertical coma between both groups were not significant (*P* > 0.05). The ablation was significantly associated with the post-operative increase in total HOAs, spherical aberration and horizontal coma (*P* < 0.05),but not with vertical coma (*P* > 0.05).

**Conclusion:**

Our results indicate that using the Triple-A ablation profile of the MEL 90 excimer laser associated with thin-flap is a safe, efficient, and predictable method to correct SE up to − 11.15D. However, for patients with high myopia, under the premise of ensuring a certain optical zone diameter, the ablation depth should be minimized to reduce the increase of the post-operative HOAs so as to improve the visual quality.

## Background

Myopia is the most common refractive disorder. It is predicted that myopia and high myopia account for 49.8 and 9.8% of the world’s population by 2050 respectively [1]. Patients with extreme myopia usually refer to those patients with SE exceeding − 9.00 D. To some extent, extreme myopia affects the patient’s athletic ability, daily life and work. At present, surgery has become a better choice for high myopia correction. The current surgical procedures for high myopia include corneal refractive surgery and posterior phakic IOL surgery. Numerous studies have shown that both operations can achieve significant improvement in high myopia correction [2]. Laser in suit keratomileusis (LASIK) is a safe procedure with predictable results for high or low myopia correction, but the outcomes for extreme myopia is not satisfactory [3]. FS-LASIK has been developed rapidly for myopia correction with high accuracy and predictability in flap thickness creation [4]. It reduces the complications of flap making and makes ultra-thin corneal flap accurately, especially in the treatment of high myopia [5, 6]. However, whether the FS-LASIK surgery could achieve better results for extreme myopia is an important issue, especially in term of postoperative visual quality and iatrogenic keratectasia [7]. The Triple-A ablation profile (Carl Zeiss Meditec, Jena, Germany) combining minimal ablation depth and spherical aberration, guarantees excellent clinical outcomes for patients with high or low myopia [8–10]. Theoretically, the surgical procedure of combining femtosecond thin- flap and the Triple-A profile should be able to correct extreme myopia. Therefore, we conducted a prospective study to investigate the efficacy and safety of thin- flap FS-LASIK with the Triple-A profile for extreme myopia correction.

### Patients and methods

In this prospective study, 90 eyes of 90 patients received FS-LASIK were enrolled at Affiliated Hospital of Nanjing University of Chinese Medicine, from January to July, 2016. Based on the SE, patients were divided into two groups: first group (ranged from − 6.00 D to − 9.00D) and second group (ranged from − 9.00D to − 11.15D). This study was approved by the Ethics Committee of our institution and adhered to the tenets of the Declaration of Helsinki. An informed consent was obtained from each of the patients after explaining the nature of the study. The inclusion criteria included the study subjects over the age of 18 with stable refraction for at least two years, regular corneal topography, and no history of ocular surgery. Patients were required to follow up cotinuously for 6 months after surgery. All surgeries were performed by the same surgeon (LK).

### Data collection

All patients underwent complete ophthalmic examinations, including the uncorrected distance visual acuity (UDVA), corrected distance visual acuity (CDVA), slitlamp microscopy, intraocular pressure measurement with non-contact tomometre (NCT), dilated indirect fundoscopy, and Pentacam Imaging examinations (Oculus, Wetzlar, Germany). Measurement of corneal higher-order aberrations (HOAs) and elevation of the posterior central elevation (PCE) were obtained using Pentacam in a dark room. The root mean square (RMS) of the 4th order spherical aberration, 3rd coma and total higher order aberrations were calculated. The ΔPCE was defined the difference between the post-operative data and the pre-operative data. All patients have been followed for 6 months. Post-operative examinations were conducted at 1 day, 1 week, and 1, 3 and 6 momths after surgery.

### Surgical procedure

The Carl Zeiss Refractive Suite, specifically the Visumax femtosecond and MEL 90 excimer lasers (Carl Zeiss Meditec, Jena, Germany), was employed for all FS- LASIK procedures. All patients had planned flap with the 500-kHz femtosecond laser. The flap parameters were as follows: 8.1 mm flaps diameters, 90 um flap thicknesss, 90°side-cut angles, and 3.0 mm hinge length set in a superior orientation. After the flap was lifted, ablations were performed using the MEL-90 excimer laser with a tissue-saving function (triple-A profile). After surgery, the post-operative topical antibiotic was applied 4 times per day for 7 days and a topical steroid 4 times per day for 2 weeks, and a non-preservative tear supplement was prescribed for 1 month.

### Statistical analysis

Date were analyzed using SPSS software (ver. 18; SPSS, Chicago, IL, United States) and presented as mean ± standard deviation (SD). The normality of all data samples was performed using the Kolmogorov-Smirnov test. Independent-sample t test and chi-square test were used to compare the differences between baseline characteristics. The independent-sample t test was used for comparisons between the first group and second group. The association between the ablation depth and the variation of HOAs was evaluated using Pearson correlation coefficient. *P* < 0.05 was considered as statistically significant difference.

## Results

### Study population

The pre-operative demographic data are listed in Table 1. No significant differences were observed in age, sex, pre-operative UDVA, CDVA, and central corneal thicknesses between the first and second groups except with pre-operative SE.

**Table 1:** Preoperative demographics of the study population (mean ± SD and range)

Characteristics	Second group	First group	*P* value
No. of eyes	43	47	–
Age, y (range)	24.3 (17~ 40)	22.5 (17 ~ 39)	0.163*
Male/Female	23/20	23/24	0.663†
Preoperative SE	−9.50 ± 0.58D (− 9.00 ~ − 11.15D)	− 7.14 ± 0.90D (− 6.00 ~ − 8.75D)	0*
UDVA (LogMAR)	1.544 ± 0.237 (1.001~2.003)	1.504 ± 0.123 (1.302~1.701)	0.336*
CDVA (LogMAR)	0.033 ± 0.035 (−0.001~0.073)	0.015 ± 0.039 (− 0.001~0.050)	0.091*
Corneal Thickness (um)	548.8 ± 18.1 (492~586)	545.5 ± 20.5 (492~583)	0.098*

UDVA: uncorrected distance visual acuity. CDVA: corrected distance visual acuity. D: diopters

* independent- sample t test

†chi-square test

### Refractive outcomes

**Fig. 1:**
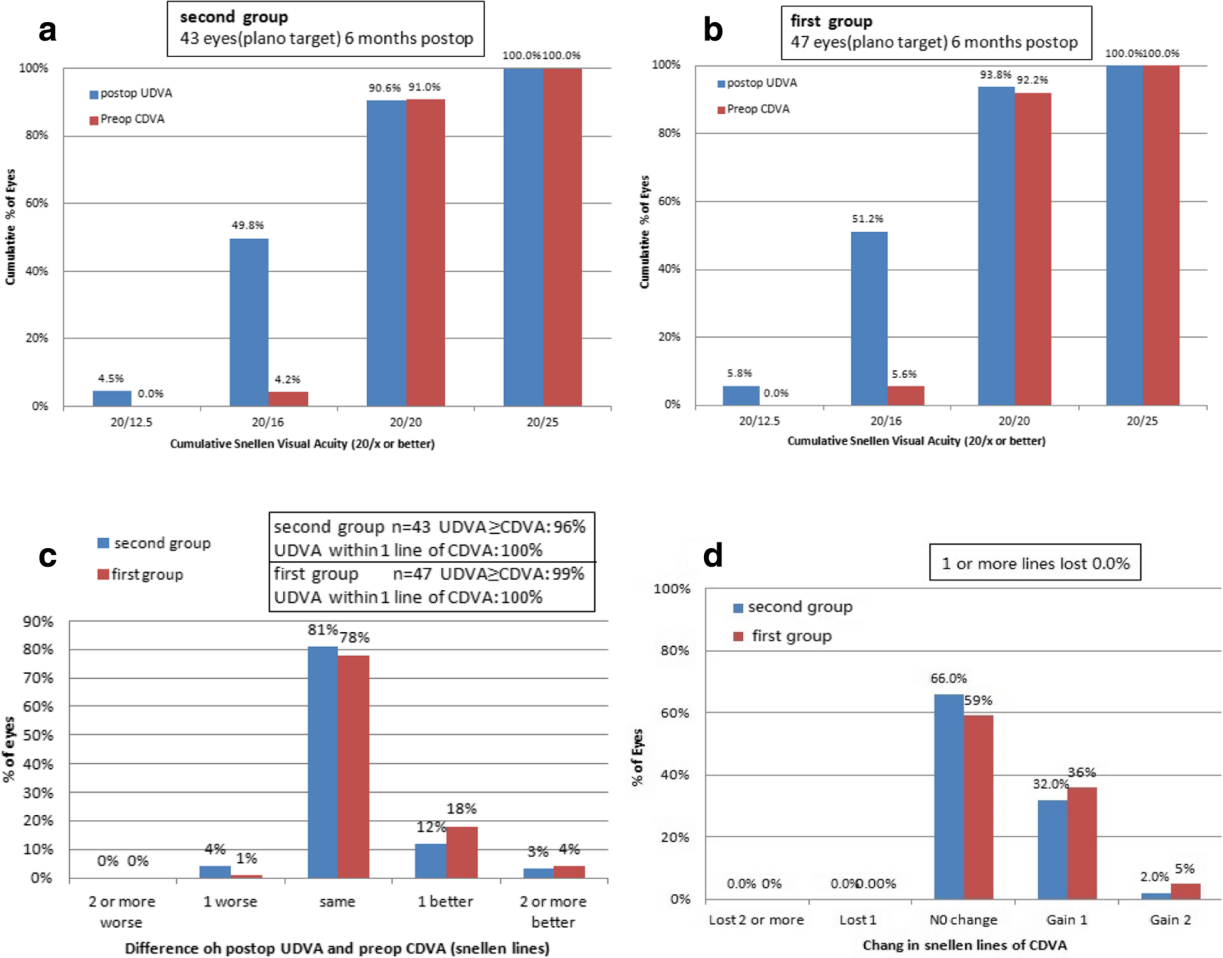
Visual outcomes at 6 months: Cumulative percentage of eyes with preoperative CDVA and postoperative UDVA result (**a** and **b**), percentage of eyes with various changes in postoperative UDVA and preoperative CDVA (**c**), and various changes in CDVA (**d**)

Table 2 displayed the efficacy and safety index of 6 months after surgery, the index for the first group were 1.149 ± 0.150 and 1.135 ± 0.154, respectively, whereas the index for the second group were 1.173 ± 0.136 and 1.158 ± 0.137 respectively. There were no significant difference between the two groups (*P* > 0.05). Figure 1 shows the distribution of UDVA and CDVA after surgery between the both groups. At sixth months after surgery, 90.6 and 49.8% of eyes in the second group had a UDVA of 20/20 and of 20/16, respectively (Fig. 1a). In the first group, 93.8 and 51.2% of the eyes had a UDVA of 20/20 and of 20/16, respectively (Fig. 1b). There were no significant differences between the both groups at the 20/20 and the 20/16 levels(χ^2^ = 0.264, *P* = 0.607 and χ^2^ = 0.045, *P* = 0.833, respectively). The gain–loss data (pre-operative CDVA versus post-operative UDVA) showed that no eye lost more than one Snellen line of CDVA in either group (Fig. 1c). In contrast, 34% of the eyes in the second group and 41% of the eyes in the first group showed a gain of Snellen lines (Fig. 1d), but they showed no statistically significant difference between both groups (χ^2^ = 0.599, *P* = 0.439).

**Table 2:** Comparison of the efficacy and safety index between second group and first group (mean ± SD)

Group	Eyes	Efficacy index	Safety index
First group	47	1.149 ± 0.150	1.135 ± 0.154
Second group	43	1.173 ± 0.136	1.158 ± 0.137
t		−1.317	−1.291
*P*		0.188	0.197

**Fig. 2:**
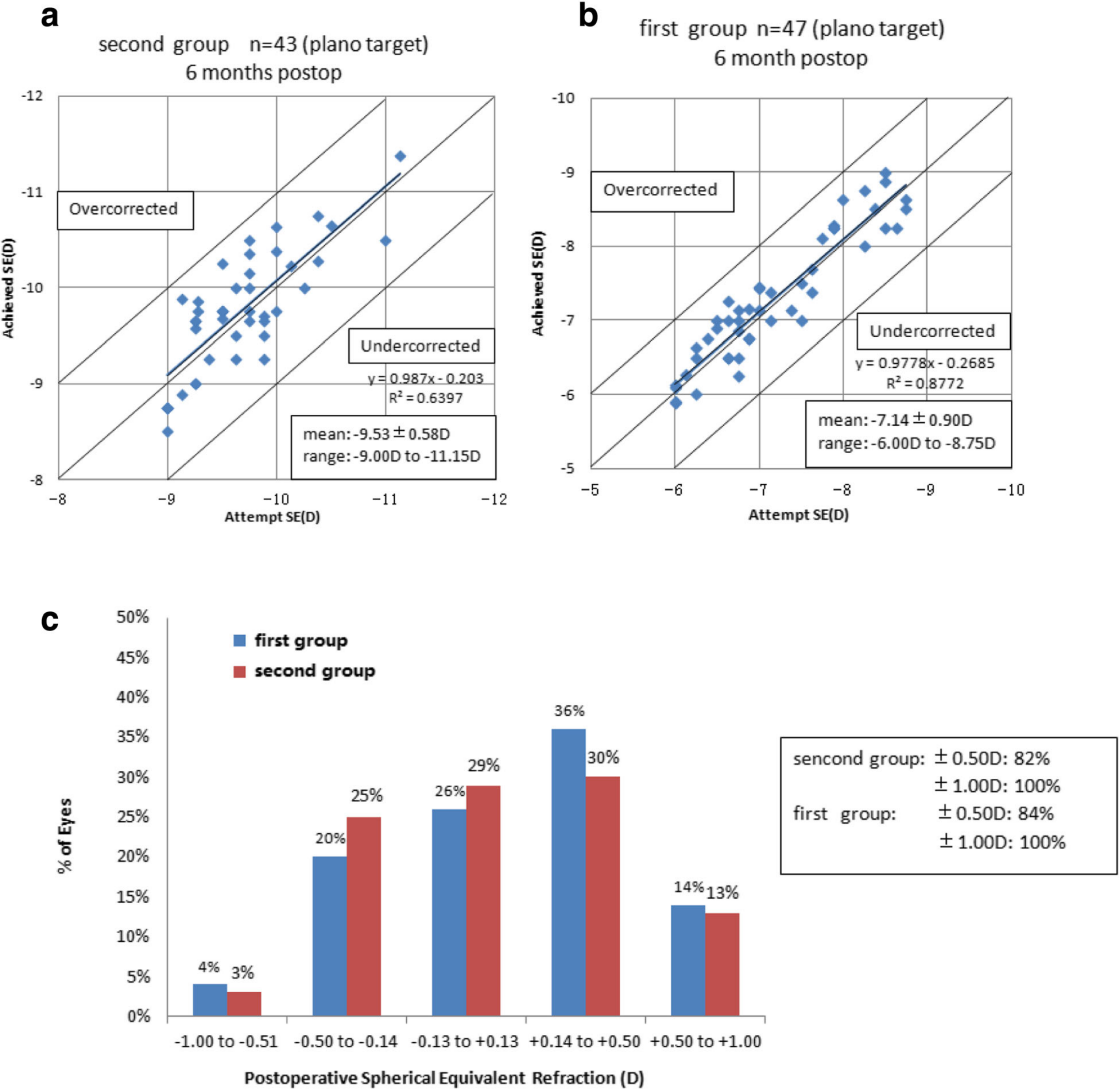
Scatterplot of the attempted versus achieved manifest spherical equivalent (SE) correction at 6 months postoperatively in both groups (**a** and **b**). Refractive outcomes at 6 months: Percentages of eyes within different diopter ranges of the intended correction in SE (**c**)

Figure 2a and b showed the scatter plots of the attempted versus the achieved SE correction at post-operative 6- month follow up. The percentage of the post-operative SE was shown in Fig. 2c that 82% (35 eyes) and 100% (43 eyes) in the second group were within ±0.50 D and ± 1.00 D of the attempted correction respectively, 84% (39 eyes) and 100% (47 eyes) in the first group were within ±0.50 D and ± 1.00 D respectively.

**Fig. 3:**
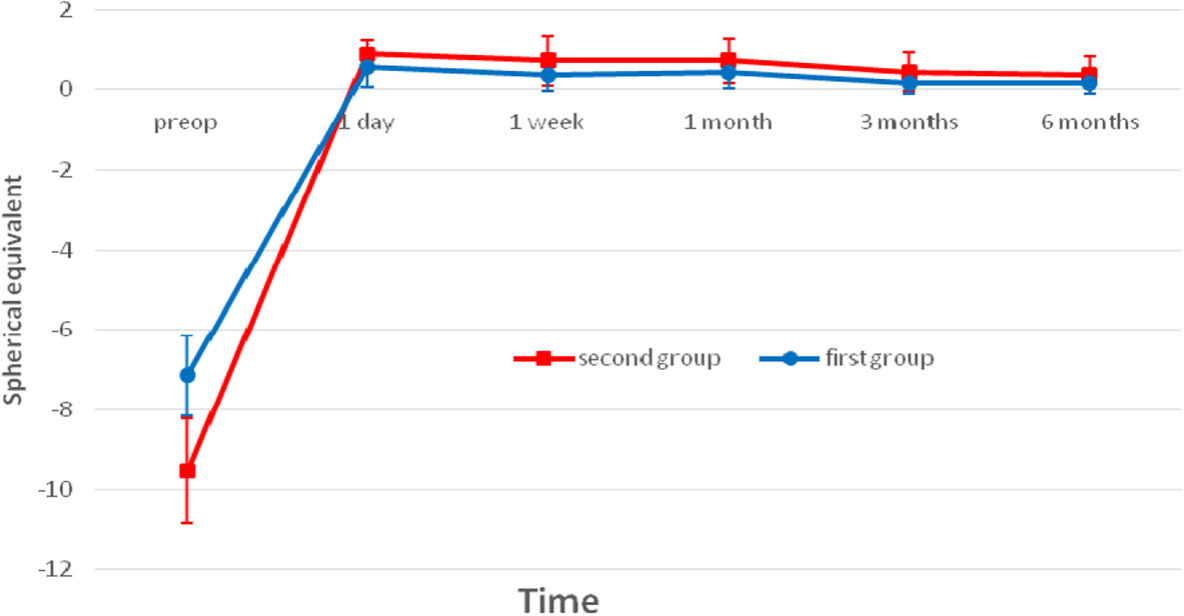
Stability of spherical equivalent refraction

Figure 3 showed the trend of mean post-operative SE in both groups. At post-operatively 6-month follow up, the SE in the first and second groups were 0.15 ± 0.26 D and 0.36 ± 0.46 D, respectively. There was no significant myopia regression in both groups after 6- month follow-up.

### Posterior central elevation (PCE) outcomes

**Fig. 4:**
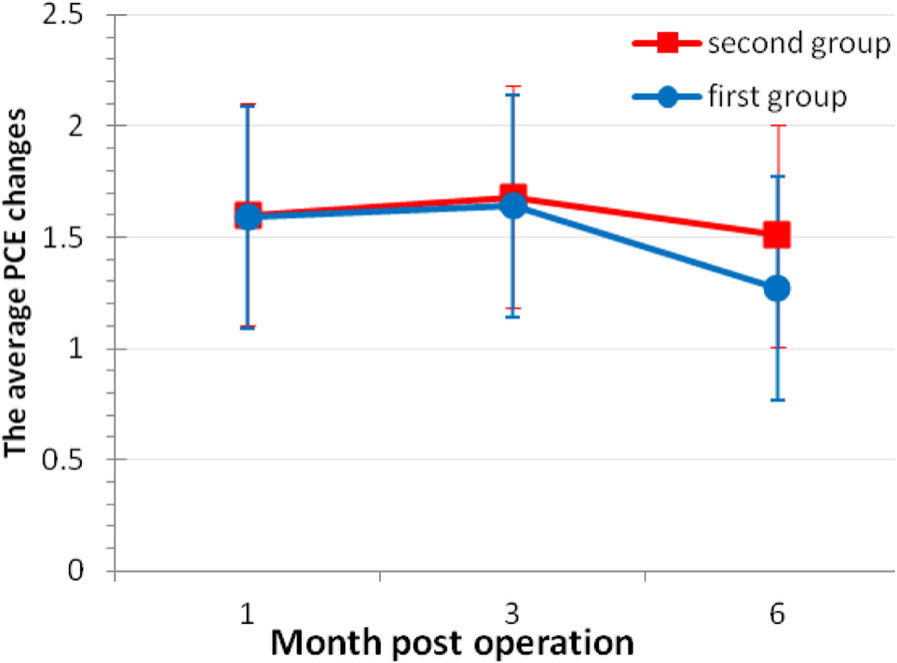
Changes in posterior central elevation (ΔPCE) at 1 month, 3 months, 6 months post operation in two groups

As shown in Fig. 4, the average PCE changes (ΔPCE) at 1, 3, and 6- month post-operative follow- up in the second group were 1.60 ± 1.00 μm, 1.68 ± 1.22 μm, 1.51 ± 1.07 μm respectively. The average ΔPCE in the first group were 1.59 ± 1.11 μm, 1.64 ± 1.19 μm, 1.27 ± 0.71 μm, respectively. One-way repeated measures ANOVA test showed no significant difference in PCE change over the 1, 3 and 6- month post-operative period for either the second group (F = 0.251, *P* = 0.778) or the first group (F = 1.705, *P* = 0.186).

### Corneal higher-order aberrations (HOA)

Table 3 displayed that the values of HOA increased significantly in both groups after surgery. The induction of total HOAs, spherical aberration, and horizontal coma in the first group were significantly less than that in the second group at 6- month follow-up post-operatively (*P* = 0, 0, 0.019 respectively), while the RMS value of vertical coma between both groups were no significantly difference (*P* = 0.927).

**Table 3:** Comparison of aberration before and after surgery between second group and first group (mean ± SD)

Groups	n	Total HOAs		Spherical aberration		Horizontal coma		Vertical coma	
		Pre-op	Post-6mo	Pre-op	Post-6mo	Pre-op	Post-6mo	Pre-op	Post-6mo
First group	47	0.344 ± 0.088	0.479 ± 0.078	0.204 ± 0.071	0.348 ± 0.124	0.205 ± 0.119	0.331 ± 0.130	0.043 ± 0.026	0.053 ± 0.045
Second group	43	0.371 ± 0.136	0.604 ± 0.103	0.221 ± 0.040	0.437 ± 0.100	0.235 ± 0.139	0.409 ± 0.182	0.044 ± 0.021	0.054 ± 0.022
t		1.126	6.495	1.494	3.716	1.094	2.391	0.225	0.092
*p*		0.264	0.000	0.140	0.000	0.277	0.019	0.822	0.927

### Correlation between the ablation depth and the variation of HOA

The tissue ablation was significantly correlated with the post-operative increases of total HOAs (r = 0.426, *P* = 0.000), spherical aberration (r = 0.243, *P* = 0.021) and horizontal coma (r = 0.341, *P* = 0.001),but not with vertical coma (r = 0.034, *P* = 0.750).

## Discussion

In recent years, corneal refractive surgery is a preferred method for myopia correction, but it is not optimal for patients with extreme myopia and thin corneal thickness. The post-operative visual acuity is easy to recede and the visual quality is usually affected after the surgery [11–13]. Lin et al. proposed the concept of thin flap LASIK and concluded that its combination with ultra-thin corneal flap cutting enables LASIK safely [14]. The thin-flap FS-LASIK not only increases the range of refractive correction, but also preserves the stromal bed thickness and increases the safety and stability of the surgery. The effect of FS- LASIK on low, medium and high myopia has been affirmed, but there are only few studies reporting the effect of extreme myopia [15–17]. MEL 90 excimer laser provides Triple-A ablation mode, which adopts the compensation algorithm of enhanced energy correction and integrates the cutting mode of aspherical and conserving corneal tissue. This new profile combines the Tissue Saving Ablation (TSA) profile with the Aberration Smart Ablation (ASA) profile, which better visual outcomes can be obtained in myopia correction, not only inducing less HOAs, but also reducing the ablation depth [9, 18]. Reinstein et al. found that it achieved high safety and efficacy for sphere up to − 10.00 D and cylinder up to 5.00 D [10]. Therefore, it is feasible to correct extreme myopia with the surgical mode of combining the Triple-A profile of excimer laser and femtosecond thin-flap.

In the present study, we found that the UCVA of the two groups of patients has been significantly improved after surgery, indicating that the correction effect is ideal. The results of this study showed that the safety index and effectiveness index of each time point for the extreme myopia group were all > 1 and no eyes demonstrated a loss in CDVA, indicating that FS-LASIK using the Triple-A profile has good safety and efficacy for high and extreme myopia correction. Another safety indicator for corneal refractive surgery is the change in the height of the PCE. Because the corneal integrity is destroyed after laser ablation, its biomechanical stability is lower than that before surgery, possibly leading to corneal posterior surface changes under intraocular pressure [19, 20]. We investigated the potential change in corneal stability by analyzing PCE after surgery. The results showed that there was no significant change in the PCE after surgery in both groups, which further indicated that the safety of FS-LASIK with tissue saving mode for extreme myopia correction was similar to that for high myopia correction. In terms of post-operative stability, both groups had mild hyperopia drift after surgery, and it was more prominent in the second group during the early stage. The SE of both groups tended to be stable at 3- month post-operatively. The observation at 6- month follow-up showed that the FS-LASIK using the Triple-A profile had good refractive stability in extreme myopia subjects.

Corneal refractive surgery can help patients return to normal vision, while visual quality often declines, especially in the HOAs. After LASIK, there is often a problem of decline in visual quality, mainly due to the increase in HOAs caused by corneal flap and stromal ablation [21–23]. The increase of spherical aberration is mainly related to excimer ablation, while the increase of coma is related to the decentration of ablation [24]. Previous studies have shown that applying femtosecond laser to create a corneal flap produces less HOAs than the mechanical microkeratome [25, 26]. In the present study, the induction of post-operative total HOA, spherical aberration and horizontal coma were significantly more obvious for eyes in extreme myopia group than for those in high myopia group, while there was no significant difference in vertical coma between the two groups. These results were in line with the correlation between the ablation depth and the variation of HOAs. With the increase of corneal tissue ablation depth, the amount of HOAs were increased after operation, showing a positive correlation pattern between them. The results indicated that the ablation depth should be minimized under the premise of ensuring a certain optical zone diameter, so as to reduce the increase of HOAs after operation.

## Conclusions

In summary, the surgical mode of combining femtosecond thin-flap and the Triple-A profile of excimer laser showed comparable results in terms of efficacy, safety, and stability for extreme myopia (SE up to − 11.15D) and high myopia. However, for patients with high myopia, under the premise of ensuring a certain optical zone diameter, the ablation depth should be minimized to reduce the increase of post-operative HOAs. Further studies with larger cohort sizes and longer-term of follow-up should be required to corroborate these findings.

## Abbreviations

CCT: Central corneal thickness
CDVA: Corrected distance visual acuity
FS-LASIK: Femtosecond laser-assisted LASIK
HOAs: High-order aberrations; RMS: root mean square
LASIK: Laser-assisted in situ keratomileusis
logMAR: Logarithm of minimum angle resolution
SE: Spherical equivalent
UDVA: Uncorrected distance visual acuity
